# Sarcopenia and frailty among older Chinese adults: Findings from the CHARLS study

**DOI:** 10.1371/journal.pone.0312879

**Published:** 2024-11-07

**Authors:** Weiwei Xu, Jiasheng Cai, Yichen Liu, Lian Li, Xiaomei Ye, Ping Wang, Jiaping Lu, Mohammed Ahmed Al-Kaif, Min Zhang

**Affiliations:** 1 Department of Endocrinology and Metabolism, Qingpu Branch of Zhong Shan Hospital Affiliated to Fudan University, Shanghai, China; 2 Department of Cardiology, Qingpu Branch of Zhong Shan Hospital Affiliated to Fudan University, Shanghai, China; Bursa Ali Osman Sonmez Oncology Hospital, TÜRKIYE

## Abstract

**Background:**

Sarcopenia and frailty are common among elderly individuals and present substantial health hazards. Exploring their relationship is essential for optimizing geriatric healthcare, particularly within the context of China.

**Methods:**

A population-based cross-sectional design was employed using data from the China Health and Retirement Longitudinal Study (CHARLS). The study enrolled 5,714 participants aged ≥60 years who underwent assessment for sarcopenia following the criteria established by the Asia Working Group for Sarcopenia (AWGS 2019) in 2019. Frailty status was determined using a frailty index that categorized participants into robustness, pre-frailty, and frailty stages. Multivariable logistic regression models were employed to examine the relationship between sarcopenia and frailty and pre-frailty conditions. Subgroup and interaction analyses were performed to explore the robustness of the associations between sarcopenia and frailty across different subgroups.

**Results:**

Among the participants, 1,028 (18.0%) were identified as frailty, 2,987 (52.3%) as pre-frailty, and 645 (11.3%) had sarcopenia. Sarcopenia demonstrated an independent association with higher risks of frailty (OR = 2.13, 95% CI: 1.52–2.99) and pre-frailty (OR = 1.42, 95% CI: 1.20–1.81) in the multivariable logistic analysis. Subgroup and interaction analyses consistently demonstrated significant associations across nearly all demographic and health-related subgroups.

**Conclusions:**

This study highlights that sarcopenia is significantly and independently associated with frailty and pre-frailty among older adults in China. Early detection and targeted interventions for sarcopenia are crucial to mitigate frailty and its adverse health outcomes in aging populations, emphasizing the need for tailored healthcare strategies to promote healthy aging.

## Introduction

Frailty is a prevalent condition among older adults, marked by decreased functional reserves, reduced resistance to stress, and increased vulnerability [1]. Studies have shown that frailty becomes more prevalent with age, ranging from 4% to 59% among older populations [2–4]. Older individuals with frailty are less resilient to stressors, which can lead to various adverse health outcomes such as fractures, cognitive decline, falls, disability, depression, and even mortality [5].

Sarcopenia, conversely, is a progressive skeletal muscle disorder characterized by the rapid loss of muscle mass and function [6,7]. Its prevalence is increasing, affecting up to 10% of elderly populations [8,9]. Meanwhile, sarcopenia is associated with many negative consequences, including fractures, falls, and mortality [10]. Many individuals with sarcopenia exhibit frailty, and conversely, frail individuals have a higher risk of sarcopenia. Sarcopenia and frailty share multiple common etiological factors, clinical characteristics, and are both interrelated conditions. Previous studies exploring the associations between sarcopenia and frailty among patients with chronic conditions, and demonstrating sarcopenia may increase the risk of frailty [11–14]. However, due to overlapping criteria and shared causes, the evidence regarding the relationship between sarcopenia and frailty among older Chinese populations remains scarce [15,16]. Clarifying this association can provide a basis for developing strategies to prevent and manage frailty effectively.

Therefore, this study aimed to explore the relationship between sarcopenia and frailty in older Chinese individuals, with the goal of increasing awareness of frailty and advocating for early interventions among those at risk.

## Methods

### Study population

The CHARLS is a continuous, nationally inclusive longitudinal survey conducted throughout China. High-quality data were collected via face-to-face interviews using a multistage, stratified sampling technique that ensured proportional representation based on population size. Participants underwent assessment with a standardized questionnaire covering sociodemographic factors, lifestyle behaviors, and health-related information. In the 2015 wave of CHARLS, 21,095 participants were recruited from 28 provinces throughout China. Further details on the study’s methodology are available on the CHARLS project website (http://charls.pku.edu.cn/). Prior to participation, all subjects provided written informed consent, and the Institutional Review Board of Peking University (IRB00001052-11015) approved the study. All procedures involving human subjects adhered to the guidelines of the institutional research committee and followed the principles outlined in the 1964 Helsinki Declaration [17].

**Fig 1:**
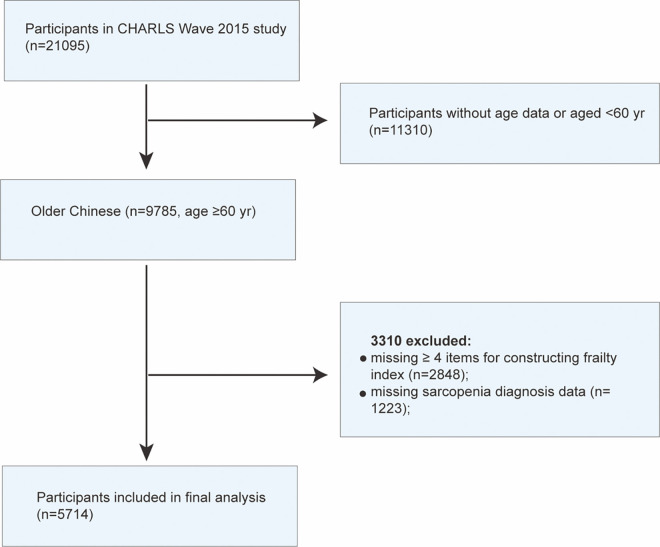
Flowchart.

Those patients with missing sarcopenia status (n = 645), incomplete age information or age < 60 years old (n = 11,310), lacking ≥ 4 or ≥ 10% items necessary for constructing the frailty index (n = 1,961) were excluded from the study. A total of 5,714 individuals were included in the final analysis, and the selection process is provided in Fig 1.

### Definitions of sarcopenia

The AWGS 2019 algorithm, which assesses three main factors muscle strength, appendicular skeletal muscle mass (ASM), and physical performance was used to determine the sarcopenia status [18]. Handgrip strength was assessed twice using a YuejianTM WL-1000 dynamometer on both dominant and non-dominant hands, with the average of the maximum measurements recorded for analysis. Prior studies have established criteria whereby individuals are classified as having low handgrip strength if their measurements fall below 28 kg for men and 18 kg for women [19]. ASM was derived using an established formula tailored for Chinese individuals [20,21], consistent with dual-energy X-ray absorptiometry.

In line with prior research findings, low muscle mass was characterized by being in the lowest 20% of height-adjusted muscle mass (ASM/height^2), specifically <4.89 kg/m^2 in women and <6.79 kg/m^2 in men [19]. Physical performance was assessed through measures such as gait speed, the chair stand test, and the short physical performance battery (SPPB), as detailed by Zhou et al. The specific criteria for defining sarcopenia have been detailed in previous publications [19]. A diagnosis of sarcopenia is made when there is a concurrent decrease in muscle mass and either poor muscular strength or poor physical performance.

### Assessment of frailty

Frailty was evaluated using the frailty index, which quantifies the accumulation of age-related health deficits. Consistent with established methodologies from prior research, the frailty index was constructed based on standardized procedures [22]. In the CHARLS dataset, a total of 36 items were utilized to formulate the frailty index, encompassing variables related to diseases, symptoms, disabilities, depression, and cognition (S1 Table). Items 1 to 34 were dichotomously coded as 0 (absence of the deficit) or 1 (presence of the deficit) using predefined cut-off points. The depressive symptoms were assessed by using the Depression Scale of the Center for Epidemiologic Studies (CESD), where depression was indicated by a score of ≥11 [23]. Item 36 was treated as a continuous variable ranging from 0 to 1, where a lower score indicated better cognitive function. When there were no missing items, the frailty index was calculated by dividing 36 by the sum of current health impairments. If the missing items < 4, the frailty index was determined by dividing the total number of valid items by the sum of their respective health impairments.

This index ranged continuously from 0 to 1, where higher values signified increased frailty. Based on criteria established in previous studies [22,24], frailty status was classified as robustness if the frailty index was 0.10 or less, pre-frailty if it ranged between 0.10 and 0.25, and frailty if 0.25 or greater.

### Covariate

Participant data encompassed a range of covariates sourced from hospital information systems and direct interviews. Variables included in the analysis encompassed age, sex, education level (categorized as primary school, middle or high school, junior college or higher), marital status (married or single), smoking status (current smoker or non-smoker), alcohol consumption (current drinker or non-drinker), residential area (rural or urban), presence of comorbidities (including hypertension, diabetes, cancer, various cardiovascular conditions such as heart attack, angina, coronary heart disease, heart failure, stroke, emotional disorders, and memory-related diseases), and blood biomarkers (such as hemoglobin, triglycerides, high-density lipoprotein cholesterol [HDL-C], low-density lipoprotein cholesterol [LDL-C], total cholesterol, creatinine, and hemoglobin A1c [HbA1c]).

The average of the three readings taken by skilled individuals during the measurement of the diastolic and systolic blood pressures (DBP and SBP) was utilized in the analysis that followed. Self-reported height and weight information collected using standardized questionnaire techniques was used to calculate the body mass index (BMI).

### Statistical analyses

Continuous data with a normal distribution are presented as mean ± standard deviation (SD), while non-normally distributed data are displayed as median with interquartile range (IQR). Categorical data are represented as counts and percentages. Group differences in continuous variables were assessed using either a one-way ANOVA or the Mann-Whitney U test, depending on the distribution of the data. Categorical variables were analyzed using the chi-squared test. Statistical significance was set at P < 0.05 for all tests. The missing values on clinical and blood biomarkers are shown in S2 Table, and multiple imputations with chained equations were employed to impute missing values for hemoglobin, triglycerides, HDL-C, LDL-C total cholesterol, creatinine, HbA1c, and 5-time chair stand test [25,26]. Meanwhile, we conducted sensitivity analyses using a complete-case analysis (*n* = 3,865) to demonstrate the stability of the study findings. Statistical analyses were conducted using R version 4.2.0 (http://www.R-project.org) and EmpowerStats software (http://www.empowerstats.com).

To assess the relationship between sarcopenia and frailty, odds ratios (ORs) and 95% confidence intervals (CIs) were calculated using both univariate and multivariate logistic regression models. Covariates were considered for inclusion in the multivariate analysis if they demonstrated a significance level of P < 0.05 in the univariate analysis. These covariates encompassed factors such as age, sex, systolic blood pressure (SBP), diastolic blood pressure (DBP), current smoking status, current alcohol consumption, residential area (urban or rural), education level, marital status, and various comorbidities including hypertension, diabetes, cancer, cardiovascular diseases (including coronary heart disease, heart attack, angina, and heart failure), stroke, emotional disorders, memory-related diseases, as well as biomarkers such as hemoglobin, triglycerides, HDL-C, and HbA1c. The selection of these covariates was also based on established associations with frailty or as previously reported in the literature [26,27]. Furthermore, subgroup and interaction analyses based on the potential confounders were performed to explore the robustness and variability of the associations between sarcopenia and frailty. Continuous variables were converted into categorical variables for subgroup analyses according to clinical or reference range cut-off points. The possible confounders were selected by previous reports as follows: age (age <75 or age ≥75 years old), gender (male or female), rural residential area, diabetes, hypertension, and cardiovascular disease [27–30].

## Results

### Baseline characteristics of selected participants

A total of 5,714 individuals participated, with an average age of 68.6 years (standard deviation 6.6 years), comprising 44.0% males. Based on the analysis, 11.3% of participants were identified as having sarcopenia. The characteristics of participants at baseline were stratified according to three categories of frailty index, as detailed in Table 1.

**Table 1:** Baseline characteristics of patients.

		Frailty index			
Characteristics	Total	Frailty	Pre-frailty	Robustness	*P*-value
		(≥ 0.25)	(0.10–0.25)	(< 0.10)	
N (%)	5714	1028	2987	1699	
Age, years	68.6 ± 6.6	70.6 ± 6.9	68.7 ± 6.6	67.1 ± 6.2	<0.001
Male, n (%)	2517 (44.0%)	355 (34.5%)	1307 (43.8%)	855 (50.3%)	<0.001
SBP, mmHg	132.5 ± 21.0	135.6 ± 22.4	132.8 ± 21.2	130.0 ± 19.4	<0.001
DBP, mmHg	74.6 ± 11.5	75.1 ± 11.8	74.7 ± 11.7	74.2 ± 10.8	0.133
BMI, kg/m^2^	24.4 ± 32.7	24.7 ± 12.3	23.9 ± 9.1	25.1 ± 57.9	0.453
Current smoker, n (%)	211 (3.7%)	22 (2.1%)	113 (3.8%)	76 (4.5%)	0.026
Current drinker, n (%)	1664 (29.1%)	184 (17.9%)	857 (28.7%)	623 (36.7%)	<0.001
Married, n (%)	4460 (78.1%)	751 (73.1%)	2299 (77.0%)	1410 (83.0%)	<0.001
Residential area, n (%)					<0.001
Urban	1409 (24.7%)	205 (19.9%)	719 (24.1%)	485 (28.5%)	
Rural	4305 (75.3%)	823 (80.1%)	2268 (75.9%)	1214 (71.5%)	
Education level, n (%)					<0.001
Illiterate	611 (10.7%)	141 (13.7%)	320 (10.7%)	150 (8.8%)	
Primary school	631 (11.0%)	103 (10.0%)	301 (10.1%)	227 (13.4%)	
Middle or high school	188 (3.3%)	22 (2.1%)	86 (2.9%)	80 (4.7%)	
Junior college or above	4284 (75.0%)	762 (74.1%)	2280 (76.3%)	1242 (73.1%)	
Comorbidities, n (%)					
Hypertension	2167 (37.9%)	594 (57.8%)	1270 (42.5%)	303 (17.8%)	<0.001
Diabetes	623 (10.9%)	228 (22.2%)	347 (11.6%)	48 (2.8%)	<0.001
Cancer	99 (1.7%)	38 (3.7%)	52 (1.7%)	9 (0.5%)	<0.001
Cardiovascular disease	1258 (22.0%)	438 (42.6%)	720 (24.1%)	100 (5.9%)	<0.001
Stroke	241 (4.2%)	110 (10.7%)	121 (4.1%)	10 (0.6%)	<0.001
Emotional problem	122 (2.1%)	53 (5.2%)	62 (2.1%)	7 (0.4%)	<0.001
Memory-related disease	203 (3.6%)	96 (9.3%)	102 (3.4%)	5 (0.3%)	<0.001
Hematological and biochemical variables					
Hemoglobin, g/dl	13.5 ± 1.9	13.4 ± 1.9	13.5 ± 1.9	13.6 ± 1.8	0.011
Triglycerides, mmol/L	1.3 [0.9, 1.9]	1.4 [1.0, 2.0]	1.3 [0.9, 1.9]	1.2 [0.9, 1.8]	<0.001
HDL-C, mmol/L	1.3 ± 0.3	1.3 ± 0.3	1.3 ± 0.3	1.3 ± 0.3	<0.001
LDL-C, mmol/L	2.7 ± 0.8	2.7 ± 0.8	2.7 ± 0.8	2.7 ± 0.7	0.556
Total cholesterol, mmol/L	4.8 ± 1.0	4.8 ± 1.1	4.9 ± 0.9	4.8 ± 0.9	0.269
Creatine, mmol/L	68.9 [58.7, 81.9]	67.7 [58.0, 82.0]	69.0 [58.4, 82.0]	69.5 [59.6, 81.5]	0.474
HbA1c, %	6.1 ± 1.0	6.3 ± 1.3	6.1 ± 1.0	6.0 ± 0.9	<0.001
Handgrip strength, kg	24.9 ± 8.7	20.5 ± 7.7	24.9 ± 8.5	27.3 ± 8.6	<0.001
ASM, kg	16.1 ± 4.3	15.4 ± 4.3	16.1 ± 4.2	16.6 ± 4.2	<0.001
5-time chair stand test, s	10.8 ± 4.4	13.1 ± 5.9	10.8 ± 4.1	9.6 ± 3.6	<0.001
Gait speed, s	3.4 [2.8, 4.2]	4.1 [3.3, 5.4]	3.4 [2.8, 4.1]	3.1 [2.6, 3.7]	<0.001
Sarcopenia, n (%)	645 (11.3%)	156 (15.2%)	349 (11.7%)	140 (8.2%)	<0.001

Notes: Mean ± SD for normally distributed continuous variables, median [Q1, Q3] for non-normally distributed continuous variables: *P*-value was calculated by one‐way ANOVA or Mann–Whitney U test. Number (%) for categorical variables: *P*-value was calculated by chi-square test.

Abbreviations: SBP: Systolic pressure; DBP: Diastolic pressure; BMI: Body mass index; HDL-C: High-density lipoprotein cholesterol; LDL-C: Low-density lipoprotein cholesterol; HbA1c: Hemoglobin A1c; ASM: Appendicular skeletal muscle mass.

Significant differences were observed across all measured characteristics between different frailty statuses, except for DBP, BMI, LDL-C, total cholesterol, and creatinine levels. Participants in the frailty group tended to be older, female, single, and residing in rural areas. They had lower educational attainment and a higher prevalence of hypertension, diabetes, cancer, cardiovascular disease, stroke, emotional problems, and memory-related disorders. They also exhibited lower levels of hemoglobin, triglycerides, handgrip strength, and ASM, alongside higher SBP, HbA1c, 5-time chair stand test, and gait speed levels. Moreover, the frailty group had the highest proportion of individuals with sarcopenia compared to the other groups.

### Association of sarcopenia with frailty and pre-frailty

In the univariate analysis, sarcopenia was found to have a positive correlation with frailty (OR = 2.13, 95% CI: 1.52–2.99, P < 0.001) and pre-frailty (OR = 1.47, 95% CI: 1.20–1.81, P < 0.001) (Table 2). Notably, other factors such as age, SBP, current drinking status, residential area, educational level, hypertension, diabetes, cancer, cardiovascular disease, stroke, emotional problems, memory-related diseases, triglycerides, HDL-C, and HbA1c also showed significant associations with frailty and pre-frailty in the univariate analysis (P < 0.05), providing a comprehensive view of our research.

**Table 2:** Association of sarcopenia with frailty and pre-frailty among older Chinese population.

	Frailty		Pre-frailty	
	Univariate analysis	Multivariate analysis	Univariate analysis	Multivariate analysis
	OR (95% CI), *P*	OR (95% CI), *P*	OR (95% CI), *P*	OR (95% CI), *P*
Age	1.08 (1.07, 1.10) <0.001	1.08 (1.06, 1.10) <0.001	1.04 (1.03, 1.05) <0.001	1.04 (1.02, 1.05) <0.001
Female	1.92 (1.64, 2.25) <0.001	1.73 (1.32, 2.26) <0.001	1.30 (1.16, 1.47) <0.001	1.17 (1.00 1.38) 0.053
SBP, mmHg	1.01 (1.01, 1.02) <0.001	1.00 (0.99, 1.01) 0.837	1.01 (1.00, 1.01) <0.001	1.01 (1.00, 1.00) 0.043
DBP, mmHg	1.01 (1.01, 1.01) 0.049	1.00 (0.98, 1.01) 0.477	1.00 (1.00, 1.01) 0.144	1.00 (0.99, 1.01) 0.750
BMI, kg/m^2^	1.00 (1.00, 1.00) 0.808	-	1.00 (1.00, 1.00) 0.366	-
Current smoker, n (%)	0.47 (0.29, 0.76) 0.002	0.48 (0.24, 0.95) 0.036	0.84 (0.62, 1.13) 0.249	0.86 (0.61, 1.21) 0.387
Current drinker, n (%)	0.38 (0.31, 0.45) <0.001	0.52 (0.39, 0.68) <0.001	0.69 (0.61, 0.79) <0.001	0.81 (0.69, 0.94) 0.007
Married, n (%)	0.45 (0.20, 1.01) 0.053	-	0.58 (0.29, 1.15) 0.121	-
Residential area, n (%)				
Urban	Ref.	Ref.	Ref.	Ref.
Rural	1.60 (1.33, 1.93) <0.001	3.35 (2.51, 4.46) <0.001	1.26 (1.10, 1.44) <0.001	1.77 (1.51, 2.07) <0.001
Education level, n (%)				
Illiterate	Ref.	Ref.	1.0	1.0
Primary school	0.48 (0.35, 0.67) <0.001	0.60 (0.39, 0.94) 0.026	0.62 (0.48, 0.81) <0.001	0.66 (0.49, 0.88) 0.004
Middle or high school	0.29 (0.17, 0.49) <0.001	0.46 (0.22, 0.97) 0.042	0.50 (0.35, 0.72) <0.001	0.58 (0.39, 0.88) 0.011
Junior college or above	0.65 (0.51, 0.84) <0.001	0.73 (0.52, 1.02) 0.064	0.86 (0.70, 1.06) 0.153	0.85 (0.68, 1.07) 0.173
Hypertension	6.31 (5.29, 7.51) <0.001	4.11 (3.22, 5.25) <0.001	3.41 (2.95, 3.94) <0.001	3.25 (2.76, 3.83) <0.001
Diabetes	9.80 (7.10, 13.53) <0.001	7.89 (5.06, 12.29) <0.001	4.52 (3.32, 6.15) <0.001	4.07 (2.88, 5.76) <0.001
Cancer	7.21 (3.47, 14.97) <0.001	7.07 (2.84, 17.59) <0.001	3.33 (1.64, 6.77) <0.001	3.94 (1.86, 8,37) <0.001
Cardiovascular disease	11.87 (9.37, 15.04) <0.001	10.74 (7.97, 14.50) <0.001	5.08 (4.08, 6.32) <0.001	4.75 (3.77, 5.99) <0.001
Stroke	20.24 (10.54, 38.85) <0.001	16.27 (7.35, 36.04) <0.001	7.13 (3.73, 13.62) <0.001	6.81 (3.47, 13.36) <0.001
Emotional problem	13.14 (5.95, 29.01) <0.001	6.14 (2.21, 17.09) <0.001	5.12 (2.34, 11.22) <0.001	4.78 (2.09, 10.91) <0.001
Memory-related disease	34.90 (14.15, 86.06) <0.001	33.68 (11.01, 103.07) <0.001	11.98 (4.87, 29.45) <0.001	9.98 (3.96, 25.17) <0.001
Hemoglobin, g/dl	0.94 (0.90, 0.98) 0.003	1.01 (0.95, 1.07) 0.767	0.98 (0.94, 1.01) 0.142	1.01 (0.97, 1.05) 0.709
Triglycerides, mmol/L	1.23 (1.13, 1.33) <0.001	1.05 (0.92, 1.20) 0.501	1.09 (1.02, 1.17) 0.009	1.06 (0.98, 1.15) 0.139
HDL-C, mmol/L	0.53 (0.41, 0.69) <0.001	0.54 (0.36, 0.82) 0.003	0.94 (0.78, 1.14) 0.526	1.06 (0.84, 1.34) 0.617
LDL-C, mmol/L	1.02 (0.93, 1.13) 0.647	-	1.05 (0.97, 1.13) 0.269	-
Total cholesterol, mmol/L	1.04 (0.97, 1.13) 0.280	-	1.05 (0.99, 1.12) 0.105	-
Creatine, mmol/L	1.00 (1.00, 1.01) 0.123	-	1.00 (1.00, 1.00) 0.100	-
HbA1c, %	1.27 (1.18, 1.38) <0.001	0.98 (0.87, 1.10) 0.681	1.14 (1.06, 1.22) <0.001	0.98 (0.90, 1.06) 0.534
Sarcopenia	1.99 (1.56, 2.54) <0.001	2.13 (1.52, 2.99) <0.001	1.47 (1.20, 1.81) <0.001	1.42 (1.13, 1.80) 0.003

Abbreviations: SBP: Systolic pressure; DBP: Diastolic pressure; BMI: Body mass index; HDL-C: High-density lipoprotein cholesterol; LDL-C: Low-density lipoprotein cholesterol; HbA1c: Hemoglobin A1c.

Upon adjusting for these confounders in the multivariate analysis, the positive association between sarcopenia and frailty or pre-frailty remained significant (frailty: OR = 2.13, 95% CI: 1.52–2.99, P < 0.001; pre-frailty: OR = 1.42, 95% CI: 1.13–1.80, P = 0.003) (Table 2). Meanwhile, we explored the associations between sarcopenia and frailty or pre-frailty using a complete-case analysis (S3 Table). The multivariate analysis demonstrated the positive and significant association between sarcopenia and frailty still existed (frailty: OR = 1.94, 95% CI: 1.25–3.02, P = 0.003; pre-frailty: OR = 1.34, 95% CI: 1.02–1.81, P = 0.044) (S3 Table).

### Relationships of sarcopenia with frailty and pre-frailty in subgroups

**Fig 2:**
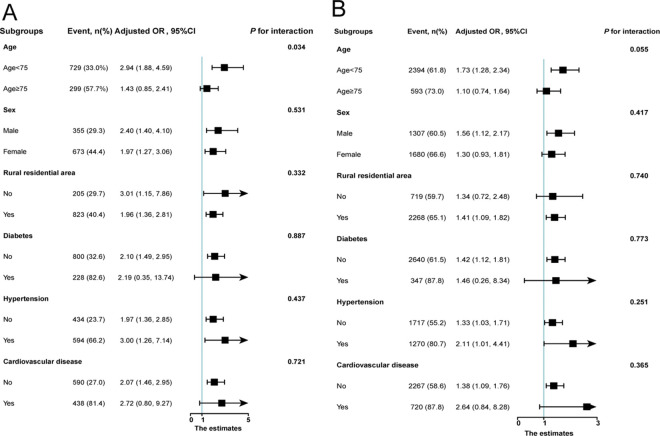
Relationships of the sarcopenia and frailty (A) and pre-frailty (B) among older Chinese population in subgroups. Notes: Adjusted OR were controlled for age, sex, SBP, DBP, current smoker, current drinker, residential area, education level married, hypertension, diabetes, cancer, cardiovascular diseases, stroke, emotional problem, memory-related disease, hemoglobin, triglycerides, HDL-C, and HbA1c. Abbreviations: SBP: Systolic pressure; DBP: Diastolic pressure; HDL-C: High-density lipoprotein cholesterol; HbA1c: Hemoglobin A1c.

We explored the associations of sarcopenia with frailty and pre-frailty across various subgroups (Fig 2). The impact of sarcopenia on frailty and pre-frailty remained consistent across almost all of these subgroups. Significant and positive associations between sarcopenia and frailty were observed among the following subgroups: age < 75 years, males and females, urban or rural residents, participants without diabetes, participants with or without hypertension, and participants without cardiovascular disease. Similarly, significant and positive associations between sarcopenia and pre-frailty were identified in subgroups including age < 75 years, males, rural residential areas, participants with diabetes, participants with or without hypertension, and participants without cardiovascular disease. However, there was an interaction observed between age groups (< 75 years and ≥ 75 years) in relation to the association between sarcopenia and frailty (P for interaction < 0.05). Specifically, the analysis indicated that participants aged < 75 years had a notably higher odds ratio (OR) for frailty when sarcopenia was present.

## Discussion

Frailty and sarcopenia are increasingly prevalent due to global population aging. This study represents the first investigation into the impact of sarcopenia on frailty and pre-frailty among Chinese adults aged 60 years and older. In this nationwide cross-sectional study, we identified that individuals with sarcopenia independently faced a heightened risk of both frailty and pre-frailty, even after adjusting for potential confounding factors. Subgroup and interaction analyses consistently underscored these significant associations across nearly all subgroup categories.

Recognition of frailty is gaining prominence in the clinical care of older individuals. Consistent with previous research [12,22], we constructed a frailty index using established procedures. Frailty was defined by a frailty index score of ≥ 0.25, identifying 18.0% of elderly adults in our study as frail. Contrasting findings from the CHARLS survey conducted in 2011–2012 (13.4% prevalence) and a pooled analysis (24% prevalence) underscored variations in frailty prevalence among older populations [31]. A multinational survey further highlighted an overall frailty prevalence of 15.2% among individuals aged 65 years and older, with regional variances ranging from 5.4% to 21.5% [32]. Variations in reported frailty rates can be attributed to differences in age demographics, underlying health conditions, genetic predispositions, and methodological approaches across studies. Research has indicated that estimates derived from frailty indices often exceed prevalence rates obtained from physical frailty phenotypes or other assessment methods [31,33]. This disparity is likely due to the frailty index’s comprehensive evaluation of health deficits, including clinical and functional manifestations, independence in daily activities, cognitive function, mobility, chronic diseases, geriatric syndromes, and depression [34]. In contrast, other methodologies typically focus on discerning specific signs and symptoms of frailty and disability. Therefore, the frailty index offers a multidimensional approach to assessing frailty, encompassing various aspects of health and functioning [35].

Recent studies have reported an incidence rate of sarcopenia in older adults at 8.5%, slightly lower than our findings [19]. This variation could be attributed to different diagnostic criteria for sarcopenia and the diversity among the enrolled adult populations [36]. Sarcopenia and frailty are both considered geriatric syndromes, sharing commonalities in mechanisms, pathophysiology, and clinical outcomes [36,37]. Sarcopenia, which primarily affects the neuromuscular system, is relatively better understood than frailty. It is characterized by a gradual decline in muscle strength and mass. It predominantly affects older adults and contributes to increased risks of fatigability, gait disturbances, balance deficits, and falls. In contrast, frail older individuals are characterized by vulnerability, diminished reserve capacity, and heightened risks of malnutrition, institutionalization, and mortality [38,39]. Research on sarcopenia typically emphasizes physical interventions aimed at enhancing muscle strength and mass, whereas frailty management often focuses on nutritional interventions and weight management. Although few studies have explored the associations between sarcopenia and frailty in older adults, there is evidence suggesting that sarcopenia and frailty may be linked to an increased risk of adverse outcomes, particularly in patients with chronic heart failure, kidney disease, and respiratory conditions [11–13]. Salgado et. Al [40] reported that sarcopenia and frailty were interrelated conditions in patients with multiple comorbidities, with most sarcopenic patients exhibiting frailty. In our study, we found that sarcopenia is associated with both pre-frailty and frailty conditions among older Chinese adults, which was consistent with recent studies conducted in the middle-aged and elderly population [14]. Therefore, interventions targeting sarcopenia management, such as dietary adjustments, physical exercise programs, and nutritional supplements, may benefit and improve frailty status [41].

Our findings demonstrated that sarcopenia increased the risk of frailty and pre-frailty among older Chinese adults. There are several hypotheses here. Firstly, frailty, as defined by a frailty index, reflects an accumulation of deficits across multiple organ systems, which overlap with sarcopenia. This overlap suggests a possible underlying connection between sarcopenia and frailty. Tsukasa et.al [42] identified a detrimental impact of skeletal muscle atrophy on memory and cognitive impairment, suggesting that sarcopenia may accelerate the progress of Alzheimer’s disease. Additionally, the frailty index calculation also includes memory-related and depressive assessment, indicating a potential link between sarcopenia and frailty. Therefore, these older adults with sarcopenia may be at an increased risk of frailty. Secondly, the patients with sarcopenia presented with low muscle mass, low muscle strength, and low physical performance. Similarly, the older adults with frailty in our study demonstrated low handgrip strength, decreased ASM, and prolonged chair stand test time. The association between sarcopenia and frailty may be attributed to these shared characteristics [14]. Thirdly, sarcopenia has been regarded as a key component of frailty, with its underlying causal mechanisms potentially involving oxidative stress, malnutrition, physical inactivity, dysregulation of inflammatory cytokines, and muscle apoptosis, all of which are hypothesized to contribute to frailty through interconnected pathways [43].

It would be valuable to explore whether the association between sarcopenia and frailty or pre-frailty holds across different subgroups. Our findings suggest a significant association between sarcopenia and increased risk of frailty and pre-frailty across nearly all subgroups examined. Although the odds ratios (ORs) were not statistically significant among participants aged ≥ 75 years, those with diabetes, or cardiovascular diseases, all these subgroups showed ORs >1, indicating a consistent trend of sarcopenia influencing frailty risk across diverse conditions. However, it’s noteworthy that we observed a significant interaction between sarcopenia and frailty among elderly individuals aged < 75 years (P for interaction = 0.034). This suggests that age < 75 years might influence the relationship between sarcopenia and frailty, warranting caution in interpretation. Further prospective studies are essential to confirm and extend these findings. Therefore, our study provides scientific evidence supporting the notion that sarcopenia is a robust indicator for assessing the likelihood of frailty or pre-frailty among elderly Chinese adults.

Our study possessed several strengths: (1) It utilized a nationwide representative sample, enhancing generalizability to the entire Chinese population, and benefited from a large sample size unprecedented in previous research. (2) To mitigate potential confounding inherent in observational studies, we employed both univariate and multivariate logistic regression models. (3) Subgroup analyses and assessments of interaction effects were conducted to ensure robustness across diverse subgroups. However, our study also had several limitations. Firstly, while we employed an anthropometric equation to estimate muscle mass and diagnose sarcopenia, validated in Chinese adults and comparable to Dual X-ray absorptiometry or Bioelectrical impedance analysis, potential measurement errors could still exist [20,21]. Nonetheless, combining anthropometric equations with handgrip strength and gait speed assessments represents a cost-effective strategy for enhancing sarcopenia diagnosis Secondly, our study did not include screening or diagnostic confirmation of sarcopenia cases, potentially introducing bias into our estimates. Thirdly, frailty and pre-frailty were defined solely based on frailty index categories, lacking validation through additional methodologies or clinical diagnosis by healthcare professionals. While this approach is consistent with previous studies using CHARLS data, the absence of supplementary assessments limits the robustness of frailty and pre-frailty diagnoses. Finally, the cross-sectional design of our study restricts us from establishing associations rather than causal relationships.

## Conclusions

In conclusion, our study demonstrates that sarcopenia is independently and significantly associated with a heightened risk of frailty and pre-frailty among Chinese older adults aged ≥ 60 years. This underscores the need for longitudinal studies and clinical trials to elucidate potential causal relationships in future research.

## Supporting information

S1 Table The 36 items used to construct the frailty index. (DOCX)

S2 Table The missing values on clinical and blood biomarkers. (DOCX)

S3 Table Associations between sarcopenia and frailty and pre-frailty using a complete-case analysis (n = 3865). (DOCX)

S1 Data (XLSX)
