# Supplementary material for: Sarcopenia and frailty among older Chinese adults: Findings from the CHARLS study
Source: PLoS One. 2024 Nov 7;19(11):e0312879. doi: 10.1371/journal.pone.0312879 (PMC11542859; doi:10.1371/journal.pone.0312879)
Supplement: S1 Table — (DOCX) [file pone.0312879.s001.docx]

**S1 Table. The 36 items used to construct the frailty index.**

| **No** | **Description of the items** | | | **Cut-off value** |
| --- | --- | --- | --- | --- |
| 1 | Self-reported physician diagnosed hypertension | | | Yes = 1, No = 0 |
| 2 | Self-reported physician diagnosed dyslipidemia | | | Yes = 1, No = 0 |
| 3 | Self-reported physician diagnosed diabetes | | | Yes = 1, No = 0 |
| 4 | Self-reported physician diagnosed heart disease | | | Yes = 1, No = 0 |
| 5 | Self-reported physician diagnosed stroke | | | Yes = 1, No = 0 |
| 6 | Self-reported physician diagnosed cancer | | | Yes = 1, No = 0 |
| 7 | Self-reported physician diagnosed arthritis | | | Yes = 1, No = 0 |
| 8 | Self-reported physician diagnosed chronic lung disease | | | Yes = 1, No = 0 |
| 9 | Self-reported physician diagnosed liver disease | | | Yes = 1, No = 0 |
| 10 | Self-reported physician diagnosed kidney disease | | | Yes = 1, No = 0 |
| 11 | Self-reported physician diagnosed gastric disease | | | Yes = 1, No = 0 |
| 12 | Self-reported physician diagnosed asthma | | | Yes = 1, No = 0 |
| 13 | Self-reported physician diagnosed any emotional, or psychiatric problems | | | Yes = 1, No = 0 |
| 14 | Self-reported physician diagnosed memory-related disease | | | Yes = 1, No = 0 |
| 15 | Self-reported eyesight (while using lenses if appropriate) |  | | Yes = 1, No = 0 |
| 16 | Self-reported hearing (while using hearing aid if appropriate) | |  | Yes = 1, No = 0 |
| 17 | Self-reported general health status | | | Poor or fair = 1, excellent, very good, or good = 0 |
| 18 | Difficulty with dressing | | | Yes = 1, No = 0 |
| 19 | Difficulty with bathing or showering | | | Yes = 1, No = 0 |
| 20 | Difficulty with eating | | | Yes = 1, No = 0 |
| 21 | Difficulty with getting in and out of bed | | | Yes = 1, No = 0 |
| 22 | Difficulty with using the toilet | | | Yes = 1, No = 0 |
| 23 | Difficulty with managing money | | | Yes = 1, No = 0 |
| 24 | Difficulty with taking medications | | | Yes = 1, No = 0 |
| 25 | Difficulty with shopping for groceries | | | Yes = 1, No = 0 |
| 26 | Difficulty with preparing meals | | | Yes = 1, No = 0 |
| 27 | Difficulty with doing housework | | | Yes = 1, No = 0 |
| 28 | Mobility: difficulty with walking 1km | | | Yes = 1, No = 0 |
| 29 | Mobility: difficulty with getting up from a chair after sitting for long periods | | | Yes = 1, No = 0 |
| 30 | Mobility: difficulty with climbing several flights of stairs without resting | | | Yes = 1, No = 0 |
| 31 | Mobility: difficulty with lifting or carrying weights over 10 pounds/jins | | | Yes = 1, No = 0 |
| 32 | Mobility: difficulty with picking up a coin from the table | | | Yes = 1, No = 0 |
| 33 | Mobility: difficulty with stooping, kneeling, or crouching | | | Yes = 1, No = 0 |
| 34 | Mobility: difficulty with reaching arms above shoulder level | | | Yes = 1, No = 0 |
| 35 | Depression: CESD-10 questionnaire | | | CESD-10 >10 =1, CESD-10 ≤10 =0 |
| 36 | Cognition: (memory test score + orientation test score) **/** 14 | | | Continuous, ranging from 0 to 1 |

Heart disease indicates the angina, coronary heart disease, congestive heart failure, or other heart problems.

Memory-related disease indicates Alzheimer’s disease or dementia, organic brain senility, or other serious memory impairment.

Depression is evaluated using Center for Epidemiologic Studies Depression Scale (CESD). In the CHARLS, CESD-10 is used, and the total score ranges from 0 to 30. The higher score indicates more severe depressive symptoms.

The memory score is the average of words that are not recalled in the immediate and delayed word recall tasks. The memory score ranges from 0 to 10. The orientation test comprises four questions about the day of the week, the month, the date of the month, and the year. One point is given for each wrong answer, and the range is from 0 to 4.
