# Supplementary material for: Sarcopenia and frailty among older Chinese adults: Findings from the CHARLS study
Source: PLoS One. 2024 Nov 7;19(11):e0312879. doi: 10.1371/journal.pone.0312879 (PMC11542859; doi:10.1371/journal.pone.0312879)
Supplement: S2 Table — (DOCX) [file pone.0312879.s002.docx]

**S2 Table . The missing values on** **clinical and blood biomarkers**

| **Characteristics** | **Missing** |
| --- | --- |
|  | N (%) |
| N (%) | 0 |
| Age, years | 0 |
| Male, n (%) | 0 |
| SBP, mmHg | 0 |
| DBP, mmHg | 0 |
| BMI, kg/m^2^ | 0 |
| Current smoker, n (%) | 0 |
| Current drinker, n (%) | 0 |
| Married, n (%) | 0 |
| Residential area, n (%) | 18 (0.3) |
| Education level, n (%) |  |
| Comorbidities, n (%) |  |
| Hypertension | 0 |
| Diabetes | 0 |
| Cancer | 0 |
| Cardiovascular disease | 0 |
| Stroke | 0 |
| Emotional problem | 0 |
| Memory-related disease | 0 |
| Hematological and biochemical variables | |
| Hemoglobin, g/dl | 997 (17.4) |
| Triglycerides, mmol/L | 964 (16.9) |
| HDL-C, mmol/L | 964 (16.9) |
| LDL-C, mmol/L | 965 (16.8) |
| Total cholesterol, mmol/L | 964 (16.9) |
| Creatine, mmol/L | 965(16.8) |
| HbA1c, % | 944(16.5) |
| Handgrip strength, kg | 0 |
| ASM, kg | 0 |
| 5-time chair stand test, s | 344 (6.0) |
| Gait speed, m/s | 222 (3.9) |
| Sarcopenia, n (%) | 0 |
