# Supplementary material for: Sarcopenia and frailty among older Chinese adults: Findings from the CHARLS study
Source: PLoS One. 2024 Nov 7;19(11):e0312879. doi: 10.1371/journal.pone.0312879 (PMC11542859; doi:10.1371/journal.pone.0312879)
Supplement: S3 Table — (DOCX) [file pone.0312879.s003.docx]

**S3 Table. Associations between sarcopenia and frailty and pre-frailty using a complete-case analysis (n=3865).**

|  | Univariate analysis  OR (95%CI, P) | Multivariate analysis  OR (95%CI, P) |
| --- | --- | --- |
| Frailty | 1.77 (1.26, 2.49) <0.001 | 1.94 (1.25, 3.02) 0.003 |
| Pre-frailty | 1.45 (1.12, 1.89) 0.005 | 1.34 (1.01, 1.81) 0.044 |

Abbreviations: SBP: systolic pressure; DBP: diastolic pressure; BMI: body mass index; HDL-C: high-density lipoprotein cholesterol; LDL-C: low-density lipoprotein cholesterol; HbA1c: hemoglobin A1c. The multivariate analysis controlled for age, sex, SBP, DBP, current smoker, current drinker, residential area, education level married, hypertension, diabetes, cancer, cardiovascular diseases, stroke, emotional problem, memory-related disease, hemoglobin, triglycerides, HDL-C, and HbA1c.
